# Revitalizing polycystic ovary syndrome: The therapeutic impact of low-dose ∆ tetrahydrocannabinol-9 through reduction of oxidative stress and modulation of macrophage polarization

**DOI:** 10.22038/IJBMS.2024.73892.16061

**Published:** 2024

**Authors:** Saeed Zavareh, Zavareh Mirseyyed, Meysam Nasiri, Hamid Hashemi-Moghaddam

**Affiliations:** 1 School of Biology, Damghan University, Damghan, Iran; 2 Department of Biology, Damghan Branch, Islamic Azad University, Damghan, Iran; 3 Department of Chemistry, Damghan Branch, Islamic Azad University, Damghan, Iran

**Keywords:** Cannabinoids, Macrophage inflammatory – proteins, Oxidative stresses, Polycystic ovarian, syndrome

## Abstract

**Objective(s):**

Polycystic ovary syndrome (PCOS) is a complex metabolic and endocrine disorder associated with chronic inflammation. However, the effect of ∆ tetrahydrocannabinol-9 (THC) on PCOS has not been evaluated. Therefore, this study aimed to investigate the immunomodulatory effects of THC in an animal model of PCOS.

**Materials and Methods:**

Twenty female Sprague-Dawley rats, aged 4 weeks, were divided into four groups. The control group received a normal diet, the sham group received a vehicle (carboxymethyl cellulose), the PCOS group received a high-fat diet (HFD) for 16 weeks followed by letrozole for 4 weeks, and the THC group received an HFD for 16 weeks followed by letrozole+THC (0.02 mg/kg) for 4 weeks.

**Results:**

The PCOS animals exhibited significantly higher levels of testosterone, insulin, triglycerides, and total cholesterol, along with elevated inflammatory and oxidative stress markers compared to the control group. Flow cytometry and real-time PCR analysis revealed an increase in M1 macrophage markers and a decrease in M2 macrophage markers compared to the control group. However, the administration of a low dose of THC mitigated these disturbances.

**Conclusion:**

Low-dose THC improved inflammatory responses and shifted the balance of M1/M2 macrophage markers towards M2 macrophages in the animal model of PCOS.

## Introduction

Polycystic ovary syndrome (PCOS) is a common multifactorial endocrine and metabolic disorder that affects women of reproductive age (1). Although the exact pathophysiology of PCOS remains unclear, previous studies have associated the condition with various clinical and biochemical manifestations, including oligomenorrhea, polycystic ovarian morphology on ultrasound, hyperandrogenism, central obesity, insulin resistance, oxidative stress (OS), and chronic low-grade inflammation (2).

It is suggested that the low-grade inflammation in PCOS may induce hypoxia in adipose tissue, leading to activation of the Nuclear factor kappa-light-chain-enhancer of activated B cells (NF-κB) inflammatory pathway. This, in turn, results in overexpression of pro-inflammatory cytokines and chemokines such as Tumor Necrosis Factor Alpha (TNF-α), Interleukin (IL)-6, 1β, 18, Monocyte chemoattractant protein-1 (MCP-1), chemokine (C-C motif) ligands 2 and 5. Additionally, this initial inflammation may attract monocyte-derived macrophages to the adipose tissue, thereby sustaining the inflammatory state and disrupting adipocyte function, ultimately leading to adipocyte necrosis. Dysfunction of adipose tissue contributes to the metabolic and reproductive phenotypes observed in women with PCOS (3).

Macrophages exhibit distinct functions depending on their phenotypic polarization. Based on this, activated macrophages are classified into two subtypes: M1 and M2 macrophages. These macrophage subtypes act in opposite ways by stimulating T helper (Th) 1- or 2-like responses, respectively (4). M1 macrophages overexpress markers such as the cluster of differentiation (CD)80, CD16/32, and CD86, and secrete pro-inflammatory cytokines such as TNF-α, IL-1β, IL-18, and IL-12, as well as nitric oxide and reactive oxygen intermediates, thereby promoting the inflammatory response (4-6). Conversely, M2 macrophages overexpress markers such as arginase (ARG)1, CD163, and CD206, and secrete anti-inflammatory cytokines that aid in tissue repair (4-6). Imbalances in M1/M2 macrophage polarization have been implicated in numerous diseases and inflammatory conditions (4). Therefore, developing new therapeutic strategies that control the dynamic changes in macrophage polarization and their interactions is crucial for advancing disease treatment (4).

Cannabinoids, derived from Cannabis sativa, have demonstrated potent anti-inflammatory properties by regulating cell-mediated and humoral immune responses (7-10). Phytocannabinoids, including ∆ tetrahydrocannabinol -9 (THC) and cannabidiol (CBD), have been shown to possess immunosuppressive roles (7-11). Previous studies have also demonstrated the efficacy and safety of THC and CBD in treating inflammatory disorders (7, 12-14). The significance of endocannabinoids as essential modulators of female reproductive systems is gaining attention. It has also been linked to changes in the female reproductive system, including folliculogenesis, oocyte maturation, and ovarian endocrine secretion (15). Also, it has been shown that endocannabinoids are crucial in the pathophysiology of PCOS (16). However, the effects of THC on PCOS have not been evaluated, and the modulation of macrophage phenotypes has rarely been studied. Therefore, the present study aimed to investigate the effects of low-dose THC in a rat model of PCOS, focusing on M1/M2 macrophage polarization, inflammatory properties, and oxidative status.

## Materials and Methods

***Materials***

All materials used in this study were purchased from Sigma Aldrich, unless otherwise specified in the text.

***Animals and study design***

Female Sprague-Dawley rats, 6 weeks old, weighing 180-200g (n=20), were obtained from the Pasteur Institute of Iran. The rats were housed in the animal facility at the University of Damghan, Iran, and allowed to acclimate to controlled conditions, including a 12-hour light/dark cycle, temperature was maintained at 22–24 °C, and humidity at 45±2%. All animal experiments were conducted in accordance with protocols approved by The Laboratory Animal Ethics Committee of Damghan University (IR.BSDU.REC.1399.14), following the guidelines of the declaration of Helsinki. After a 2-week adaptation period, the rats were divided into four groups, with five rats per group: control, sham, PCOS, and THC. The control group received a standard laboratory diet consisting of 3.14 kcal/g, with an energy supply ratio of 21.5% protein, 65% carbohydrates, and 4% fat, for 16 weeks. The sham group received the standard laboratory diet for 16 weeks, along with 5 ml of carboxymethyl cellulose 0.5% (CMC) administered orally through gavage as a vehicle, daily for the last four weeks. The PCOS group was fed a high-fat diet (HFD) comprising 5.3 kcal/g, with an energy supply ratio of 20% protein, 36% carbohydrates, 40% fat, and 1.25% cholesterol, along with high sugar syrup (23.1 g/L d-fructose and 18.9 g/L d-glucose), for 16 weeks (17). Additionally, these animals received letrozole (1 mg/kg) dissolved in 5 ml CMC 0.5%, administered orally through gavage, daily for the last four weeks. The THC group received HFD along with high sugar syrup for 16 weeks, letrozole (1 mg/kg) orally and THC (10 mg/kg) which was administered intraperitoneally in ethanol daily for the last four weeks. The doses were selected based on previous reports (18). The weights of the animals were measured weekly. Estrous cycles were assessed by evaluating the cellular composition of vaginal smears, following previously described methods (19). At the end of the experiment, all rats were euthanized by decapitation under deep anesthesia using ketamine (100 mg/kg) and xylazine (10 mg/kg).

***Glucose and insulin tolerance tests***

An oral glucose tolerance test (OGTT) was conducted during the final week of the experiment. After a 15-hour fasting period, blood samples were collected from the tail vein at the start of the test (time 0). Subsequently, the rats received a glucose solution (2 g/kg body weight) via gavage. Blood samples were collected from the tail vein at 30, 60, and 120 min after glucose administration to measure glucose levels using the Decont Personal Accu-check device. Furthermore, 48 hr after the OGTT, the homeostatic model assessment insulin resistance (HOMA-IR) was calculated using the following formula: fasting plasma insulin (mU/l) × fasting plasma glucose (mmol/l)/22.5. The HOMA-B index, representing β-cell function, was calculated as the product of 20 and basal insulin levels divided by the value of basal glucose concentrations minus 3.5. Additionally, the Quantitative Insulin Sensitivity Check Index (QUICKI) was determined using the formula (1/log(FI) + log(FG)), where FI represents fasting insulin expressed in µU/ml and FG represents fasting glucose expressed in mg/dl. The insulin concentration was measured using an enzyme-linked immunosorbent assay (ELISA) kit from Merck/Merck Millipore, Hungary.

***Hormone assay***

Serum levels of testosterone, progesterone, and estradiol were measured using an ELISA kit (Demeditec, Germany) in accordance with the manufacturer’s instructions. Luteinizing hormone (LH; mIU/ml) and follicle-stimulating hormone (FSH; mIU/ml) were quantified using chemiluminescent immunoassay. To ensure accurate assessment of hormonal changes, blood samples were collected from animals in different groups at comparable stages of the estrous cycle.

Lipid Profile and C-Reactive Protein (CRP) Assay: 

Serum levels of total cholesterol (TC; mmol/L), triglycerides (TG; mmol/L), low-density lipoprotein (LDL; mmol/L), and high-density lipoprotein (HDL; mmol/L) were measured using standard colorimetric methods. Serum CRP content was determined using an ELISA kit (Millipore’s MILLIPLEX® MAP Rat/Mouse CRP Single Plex, USA).

***Histological studies***

Ovarian samples were obtained from animals at comparable stages of the estrous cycle. The ovaries were sectioned serially at 5 µm intervals from the center and stained with hematoxylin and eosin (H&E). In each set of 5 sections, taken from the largest cross-sectional area, ovarian follicles at various growth phases were examined to determine the number of preantral, antral, and atretic follicles, as well as the corpus luteum. The classification of follicles followed the previously described criteria (20).  Additionally, the thickness of the largest follicular wall, including the theca and granulosa layers, was measured.

***Gene expression analysis***

Total RNA was extracted from ovaries using Trizol (Qiagen, USA) following the manufacturer’s instructions. The RNA samples were treated with DNase I (Cinnagen, Iran) to remove any residual DNA contamination. Subsequently, cDNA synthesis was performed using the RevertAid kit (Fermentas, MD, USA) according to the manufacturer’s protocol. Real-time PCR was conducted using the Rotor-Gene6000 machine (Qiagen, Germany) and RealQ Plus SYBR Green (Ampliqon, Denmark). The primer sequences were designed using AlleleID software version 7.5 (premierbiosoft, USA) as shown in Table 1. The qPCR protocols included an initial cycle at 95 °C for 15 min, followed by 40 cycles at 95 °C for 15 sec and 60 °C for 45 sec, as recommended by the MIQE guidelines (21). The *Tbp* (TATA-box binding protein) gene was used as the reference gene for normalization. The relative mRNA levels were determined using the 2^-ΔΔCT^ method. To ensure accuracy, a no-template control (NTC) tube was included for each gene in all experiments.

***Evaluation of oxidative status***

Tissue supernatant was prepared from ovarian tissue to assess the levels of total anti-oxidant capacity (TAC) and malondialdehyde (MDA), as well as the activities of superoxide dismutase (SOD), glutathione peroxidase (GPX), and catalase (CAT), following previously described methods (22, 23). The production of reactive oxygen species (ROS) in ovarian tissue was measured using a 2’,7’-dichloro-dihydro fluorescin (DCHF) probe, as previously described (23). TAC levels (mol/L) were determined using the Ferric reducing/anti-oxidant power (FRAP) method, as described in previous studies (23). The MDA level (nmol/mg) was assessed as an indicator of lipid peroxidation, following established protocols (22). GPX activity was measured by the conversion of NADPH to NADP, with changes in absorption at 340 nm recorded. SOD activity was determined by calculating the 50% inhibition of nitro blue tetrazolium reduction. CAT activity was calculated based on the absorbance change in one minute and expressed as μMol/min/mg protein. The total protein concentration in the tissue supernatant was determined using the Lowry assay method (24).

***Flow cytometry***

Flow cytometry was conducted following a previously described method (25) with some modifications. Briefly, ovarian cells were suspended in a single-cell suspension using 250 U/ml collagenase IV at 37 °C for 30 min. Subsequently, the isolated cells were prepared using the Single-Cell Dissociator DSC-400 (RWD Life Science, China) and a 70 μm nylon mesh, followed by centrifugation at 1000 rpm for 8 min. The isolated cells were then incubated with FcR Blocking Reagent (eBioscience, San Diego, CA, USA) for 15 min. After counting, the cells were stained using fluorophore-conjugated antibodies against rat CD11c, CD206, and F4/80 (Thermo Fisher Scientific, USA), following the manufacturer’s instructions. Following staining, the detection of CD11c, CD206, and F4/80-positive cells was performed using flow cytometry, and the results were evaluated based on the percentage of positive cells.

***Statistical analysis***

All experiments were performed with a minimum of five replicates. Statistical analysis was conducted using SPSS version 16 software package for Windows (SPSS Inc., Chicago, IL, USA). One-way analysis of variance (ANOVA) with Tukey’s Honestly Significant Difference (HSD) *post hoc* test was utilized. The data are presented as mean ± standard deviation (SD). A *P*-value of less than 0.05 was considered statistically significant.

## Results

***Weight changes***

**Figure 1:**
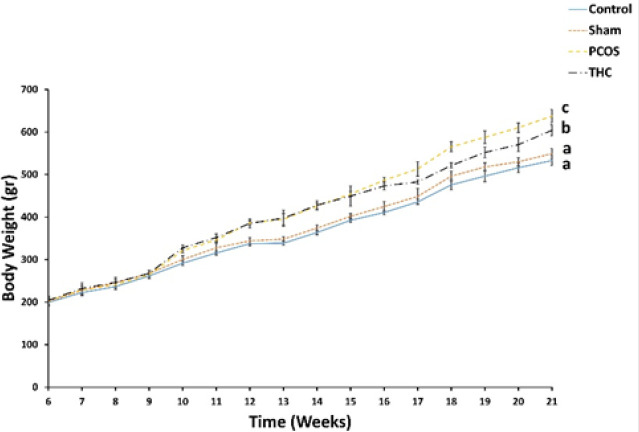
Body weight of the experimental groups measured at the 6th untill the end of experiment

At the beginning of the experiment, there were no significant differences in body weights among the experimental groups. However, starting from the fourth week, the weight of rats in the PCOS group significantly increased compared to the control and sham groups (*P*<0.001, Figure 1). There was no significant difference in weight between the sham and control groups. In contrast, the body weight of the THC-treated group decreased significantly compared to the PCOS group and was also significantly higher than the weight of the sham and control groups (*P*<0.001, Figure 1).

***Vaginal smears***

**Figure 2:**
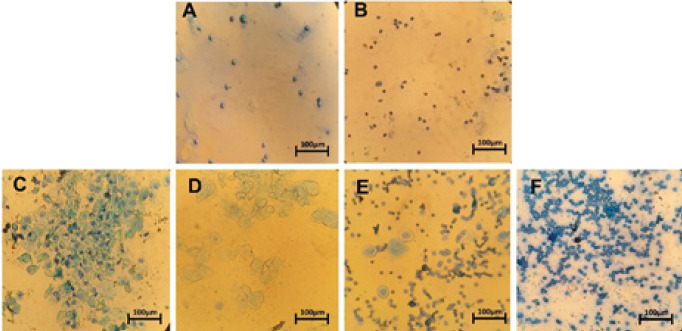
Cellular types from the vaginal smear of the experimental group, (A) Vaginal smear from a THC-treated group. (B) Vaginal smear from PCOS group. (C, D, E, and F) Vaginal smears from control and sham group with regular estrus cycle

The PCOS rats exhibited complete acyclicity, and their vaginal smears consisted mostly of leukocytes, indicating pseudo-diestrus (Figure 2). In contrast, the vaginal smears of the control, sham, and THC-treated groups showed a regular estrus cycle (Figure 2). During the proestrus phase, a few cornified epithelial cells and leukocytes with dominant nucleated epithelial cells were observed (Figure 2). The cytological appearance during the estrous phase consisted mainly of anucleate cornified epithelial cells (Figure 2). At the metestrus phase, equal proportions of leukocytes, cornified, and nucleated epithelial cells were observed (Figure 2). Lastly, a high proportion of leukocytes, some nucleated epithelial cells, and mucus were seen during the diestrus phase (Figure 2).

***Blood glucose level and insulin tolerance tests***

**Figure 3:**
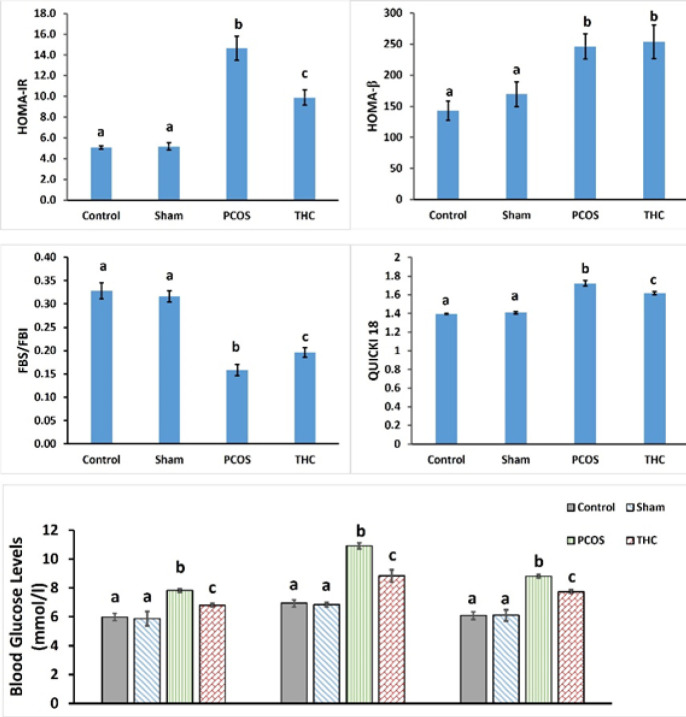
Blood glucose level and Index of insulin resistance of experimental groups

The serum blood glucose level (BGL) significantly increased in the PCOS group compared to the other groups (*P*<0.001, Figure 3). There was no significant difference in BGL between the control and sham groups (*P*>0.05, Figure 3). Treatment with THC significantly decreased BGL compared to the untreated PCOS group (*P*<0.001, Figure 3). Additionally, the homeostatic model assessment of insulin resistance (HOMA-IR) was significantly higher in the PCOS group compared to the other groups (*P*<0.001, Figure 3). In the THC-treated group, HOMA-IR significantly decreased compared to the PCOS group (*P*<0.001). However, it remained significantly higher than those of the control and sham groups (*P*<0.001). There was no significant difference in HOMA-IR between the control and sham groups. As shown in Figure 3, PCOS rats also exhibited higher QUICKI-18 levels and HOMA-β compared to the control and sham groups (*P*<0.001). However, THC treatment reduced QUICKI-18 levels compared to the PCOS group (*P*<0.001, Figure 3). Nevertheless, these levels remained significantly higher than those of the control and sham groups (*P*<0.001).

***Ovarian morphology***

**Figure 4:**
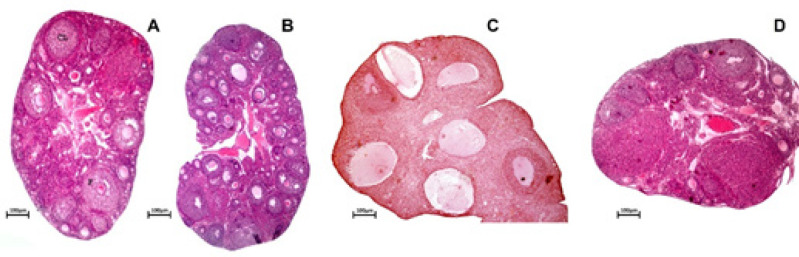
Ovarian histology, MICROGRAPHS correspond to the largest section of the stained ovary by hematoxylin-eosin

Micrographs depicting ovarian morphology, stained with hematoxylin-eosin, are presented in Figure 4. Ovarian sections exhibited no notable variations in the number of primordial and primary follicles across the experimental groups. However, the control and sham groups displayed a significantly higher count of secondary follicles compared to the other groups (*P*<0.001, Table 2). Interestingly, the THC-treated groups demonstrated a significant increase in the number of secondary follicles compared to the PCOS group (*P*<0.001, Table 2). Moreover, the PCOS groups exhibited a significantly lower count of tertiary and Graafian follicles compared to the other groups (*P*<0.001, Table 2). Although the THC-treated group showed a significant increase in the number of tertiary and Graafian follicles compared to the PCOS group (*P*<0.001), their count remained significantly lower than that of the control and sham groups (*P*<0.001).

Treatment with THC significantly increased the number of tertiary and Graafian follicles compared to the PCOS group (*P*<0.001). Additionally, there was no significant difference between the control and sham groups in terms of the number of tertiary and Graafian follicles. Ovarian sections of the control, sham, and THC groups displayed several corpus luteum (CL), whereas no CL was observed in the PCOS ovarian sections. Furthermore, the number of CL in the control and sham groups was significantly higher than in the THC-treated group (*P*<0.001, Table 2). Moreover, both the PCOS and THC groups exhibited a significantly higher count of atretic follicles compared to the control group (*P*<0.001). However, the THC-treated group had a significantly lower number of atretic follicles than the PCOS group (*P*<0.001). The control and sham groups displayed a significantly lower count of cystic follicles compared to the other groups. Conversely, the PCOS group exhibited a significantly higher mean number of total cystic follicles compared to the HFD-treated group (*P*<0.001). Additionally, the follicle wall thickness in the PCOS and THC groups was significantly increased compared to the control and sham groups, while the THC-treated groups displayed a significant decrease in follicle wall thickness compared to the PCOS group (*P*<0.001). The ovarian weight in both the control and sham groups was significantly higher than in the PCOS and THC groups (*P*<0.001). However, the ovary weight of the THC-treated group was significantly increased compared to the PCOS group (*P*<0.001, Table 2).

***Hormone assay***

**Figure 5:**
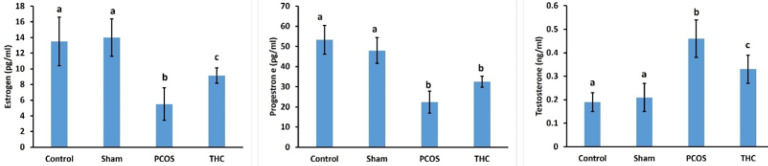
Serum levels of testosterone, estrogen, and progesterone measured using an ELISA kit (Demeditec, German)

No significant differences were observed in estrogen, progesterone, and testosterone levels between the control and sham groups. The estrogen level was significantly lower in the PCOS and THC groups compared to the control and sham groups (Figure 5; *P*<0.001). However, the estrogen level in the THC group was significantly higher than in the PCOS group (*P*<0.001). Progesterone levels were significantly decreased in the PCOS and THC groups compared to the control and sham groups (Figure 5; *P*<0.001). A similar pattern was observed in the THC and PCOS groups. Serum testosterone was significantly higher in the PCOS group compared to the other groups (Figure 5; *P*<0.001). Conversely, the testosterone level was significantly decreased in the THC group compared to the PCOS group (Figure 5; *P*<0.001).

***Lipid profile assay***

**Figure 6:**
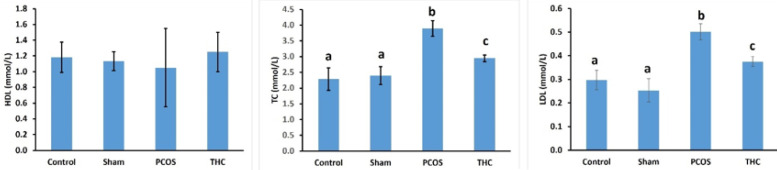
Serum levels of total cholesterol (TC; mmol/l), triglycerides (TG; mmol/l), low-density lipoprotein (LDL; mmol/l), and high-density lipoprotein (HDL; mmol/l) were measured using standard colorimetric methods

No significant difference was found among the HDL levels of the experimental groups. Total cholesterol (TC) and LDL levels in the PCOS group were significantly higher than those in the other groups (Figure 6; *P*<0.001). There was no significant difference between the TC and LDL levels of the control and sham groups (Figure 6). However, the TC and LDL levels in the THC-treated group were significantly lower than those in the PCOS group (Figure 6; *P*<0.001).

***CRP assay***

**Figure 7:**
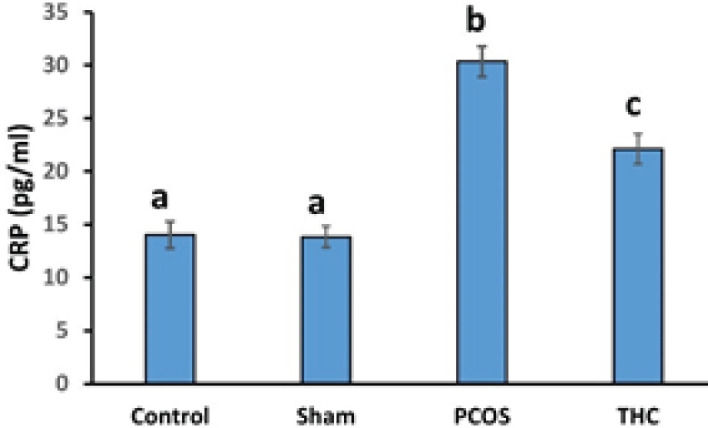
C-reactive protein (CRP) level of experimental groups

The CRP level was significantly increased in the PCOS group compared to the control and sham groups (*P*<0.001, Figure 7). Furthermore, the CRP level was significantly higher in the THC group compared to the control and sham groups (*P*<0.001, Figure 7). However, the CRP level in the THC group was significantly decreased compared to the PCOS group (*P*<0.001, Figure 7).

***Gene expression analysis***

**Figure 8:**
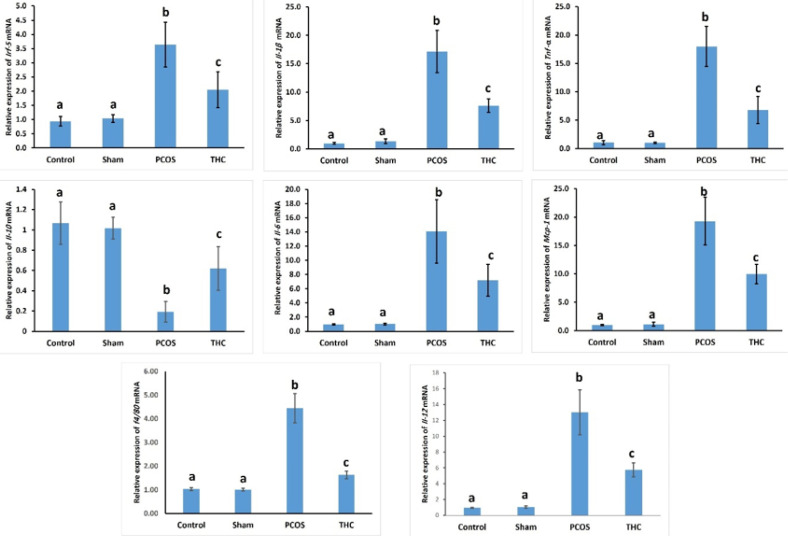
Relative mRNA expression of TNF-α, Mcp1, Il-1b, Il-6, Il-12, Irf-5, Il-10, and F4/80

The relative mRNA levels of inflammatory biomarkers such as TNF-α, Mcp1, Il-1b, Il-6, and Il-12 significantly increased in the ovarian tissue of the PCOS group compared to the control and sham groups. However, administration of THC significantly decreased these parameters compared to untreated PCOS animals (Figure 8, *P*<0.001). No significant difference was found between the control and sham groups. Additionally, there was a significant increase in the relative mRNA expression of Irf-5 and a significant decrease in the relative mRNA expression of Il-10 in the ovarian tissue of the PCOS group compared to the other groups (Figure 8, *P*<0.001). Treatment with THC led to a significant decrease in the relative mRNA expression levels of Irf-5 in the PCOS group compared to the PCOS group, while the relative mRNA expression of Il-10 increased significantly compared to the PCOS group (*P*<0.001). Furthermore, the ovarian tissue of PCOS animals exhibited a significant increase in F4/80 relative mRNA levels compared to the control and sham groups, which were significantly decreased in the THC-treated group compared to the PCOS group (Figure 8, *P*<0.001).

***Evaluation of oxidative status***

**Figure 9:**
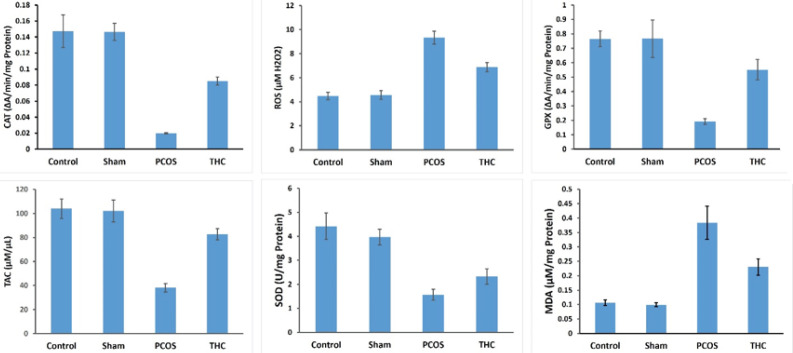
Oxidative stress parameters of ovarian tissue of experimental groups

Figure 9 illustrates the oxidative stress (OS) parameters in the experimental groups. ROS and MDA levels were significantly increased in the PCOS groups compared to the control and sham groups (*P*<0.001). Treatment with THC significantly decreased the ROS and MDA levels compared to the PCOS group (*P*<0.001, Figure 9). However, both MDA and ROS levels in the THC group remained significantly higher than in the control and sham groups (Figures 9, *P*<0.001). THC treatment also significantly increased the TAC level compared to the untreated PCOS group, although it was still significantly lower compared to the control and sham groups (*P*<0.01). The activities of SOD, GPx, and CAT, which were significantly decreased in the PCOS group compared to the control and sham groups, were significantly increased with THC treatment (Figure 9, *P*<0.001).

***Flow cytometric analysis (polarization of macrophages)***

**Figure 10:**
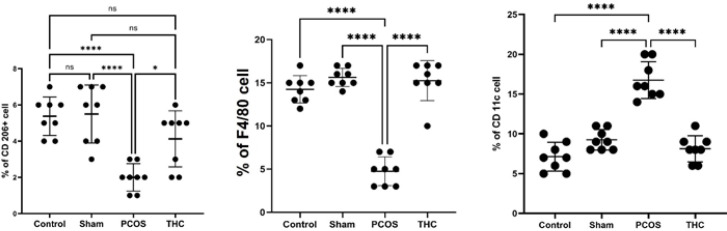
Flow cytometric analysis of macrophage bio-markers

CD11c, CD206, and F4/80 markers were used to detect M1 or M2 macrophages. CD11c+, F4/80-, and CD206- cells were considered M1 macrophages, while CD11c-, F4/80+, and CD206+ cells were considered M2 macrophages. The distribution patterns of M1 and M2 macrophages in the ovarian tissue of the experimental groups are shown in Figure 10. The number of M1 macrophages significantly increased in the PCOS group compared to the control and sham groups (*P*<0.001). However, it significantly decreased in the ovarian tissue of the THC-treated group compared to the PCOS group (*P*<0.001), although it remained significantly higher than in the control and sham groups (*P*<0.001). The number of M2 macrophages significantly increased after treatment with THC compared to the PCOS group (Figure 10, *P*<0.001).

**Table 1:** List of primers of inflammatory biomarkers gene for rat ovarian tissue

**Primers**	**Accession** **number**	**Sequence**	Product size
***Cd163-f***	NM_001107887	5´ GTGCCTCCCAAGAATGACTTTAGA ´3	127
***Cd163-R***		5´ TCGCTTCAGAGTCCACAAGA ´3	
***F4/80-F***	NM_001007557	5´ TTGGCTGCTCCTCTTCTG ´3	96
***F4/80-R***		5´ CATTCATTCACACCGTTAAGTCT ´3	
***Mcp1-F***	NM_031530	5´ ACTCATTCACTGGCAAGAT ´3	332
***Mcp1-R***		5´ TGTCATACTGGTCACTTCTAC ´3	
***Tnf-F***	NM_012675	5´ CGTGTTCATCCGTTCTCT ´3	170
***Tnf-R***		5´ AGCATCGTAGTTGTTGGAA ´3	
***Il6-F***	NM_012589	5´TCCAGCCAGTTGCCTTCT´3	91
***Il6-R***		5´GTATCCTCTGTGAAGTCTCCTCTC´3	
***Il1*** **β** ***-F***	NM_031512	5´GATGATGACGACCTGCTA´3	147
***Il-1*** **β** ***-R***		5´CACTTGTTGGCTTATGTTCT ´3	
***Il10-F***	NM_012854	5´GCTATGTTGCCTGCTCTTA´3	218
***Il10-R***		5´CCAAGTAACCCTTAAAGTCCT´3	
***Irf5-F***	NM_001106586	5´GCAATAGTGAGGTTACAGATGG´3	205
***Irf5-R***		5´TTCAGAGACAGGCATATTAGAGA ´3	
***Tbp-F***	NM_001004198	5´ ATCACTCCTGCCACACCA ´3	249
***Tbp-R***		5´ TCTGGATTGTTCTTCACTCTTGG ´3	

The body weights of each group (n = 5/group) were recorded weekly. Different letters indicate significant differences among groups (*P*<0.001). Values are mean ± SD.

PCOS: Polycystic ovarian syndrome

(C) Proestrus; (D) Estrus; (E) Metestrus; (F) Diestrus. The magnification was 400.

Different letters indicate significant differences among groups (*P*<0.001). Values are mean ± SD. PCOS: Polycystic ovarian syndrome

A, ovary from the control group. B, ovary from sham group. C, ovary from PCOS group, and D, ovary from control THC-treated group. F, normal follicle; CL, corpora lutea; C, follicular cystic. The magnification was 40X

**Table 2:** Number of ovarian follicles at different stages of development and ovarian morphology parameters (5 sections per ovary)

Groups	**Control**	**Sham**	**PCOS**	**THC**
Primordial Follicles	8.00±0.89	8.33±1.37	7.67±1.21	7.33±2.07
Primary Follicles	6.17±0.75	6.83±1.33	7.17±1.72	5.17±1.60
Secondary Follicles	3.00±0.63^a^	3.17±0.75^a^	0.50±0.55^b^	1.83±0.75^c^
Tertiary Follicles	2.17±0.75^a^	2.17±0.75^a^	0.17±0.41^b^	1.17±0.41^c^
Graafian Follicles	2.33±0.52^a^	2.67±0.82^a^	0.00±0.00^b^	1.17±0.41^c^
Corpus Luteum	4.17±1.17^a^	4.00±0.89^a^	0.00±0.00^b^	1.83±0.75^c^
Atretic Follicles	0.83±0.75^a^	0.83±0.41^a^	11.67±1.63^b^	5.33±1.86^c^
Cystic Follicles	0.50±0.55^a^	0.17±0.41^a^	11.83±1.6^b^	3.5±1.05^c^
Follicle wall thickness	61.50±7.31^a^	62.67±3.72^a^	135.83±14.8^b^	102.5±7.64^c^
Ovarian weight	0.15±0.02a	0.15±0.03a	0.25±0.02b	0.20±0.02c

Values are mean ± SD.

Different letters indicate significant differences in the same raw (*P*<0.05)

Values are expressed as Mean ± SD. Different letters indicate significant differences (*P*<0.001).

Values are expressed as Mean ± SD. Different letters indicate significant differences (*P*<0.001). PCOS: Polycystic ovarian syndrom

Values are expressed as Mean ± SD. Different letters indicate significant differences

(*P*<0.001). PCOS: Polycystic ovarian syndrome

The data are presented as relative fold changes (Mean ± SD) of three independent experiments. Different letters indicate significant differences (*P*<0.001). PCOS: Polycystic ovarian syndrome

Values are expressed as Mean ± SD. Different letters indicate significant differences (*P*<0.001).

CD11c+, F4/80+ and CD206− cells were considered M1 cells. CD11c−, F4/80+, and CD206+ cells were considered M2 cells

PCOS: Polycystic ovarian syndrom

## Discussion

There is strong evidence suggesting that increased body weight worsens hyperandrogenism and metabolic risk in PCOS (26-28). High-fat diet (HFD) has been used to create a model of metabolic syndrome (26-28). Therefore, in this study, letrozole was utilized to induce hyperandrogenism, while HFD was employed to induce metabolic syndrome, both contributing to the development of an obese, insulin-resistant PCOS model. Our observations in the rat model align with previous research findings (28, 29). However, the investigation of the effects of THC on endocrine, inflammatory, and oxidative statuses, as well as macrophage polarization in the PCOS rat model, constitutes the most significant findings.

The findings of our study demonstrated that THC treatment reduces weight gain, fasting blood sugar (FBS), insulin resistance, testosterone levels, total cholesterol (TC), low-density lipoprotein (LDL) levels, and C-reactive protein (CRP) levels. Additionally, it increases estradiol and progesterone levels. Real-time PCR evaluation revealed a significant increase in Irf5 expression, along with significant up-regulation of Tnf-α, Il-1β, Il-6, Mcp1, and F4/80 in PCOS rats. Moreover, flow cytometry results exhibited a significant increase in the percentage of CD206 and F4/80+ cells in the THC-treated group, while the percentage of CD11 cells decreased significantly.

The results of our study indicate that THC administration leads to weight loss compared to the PCOS group. However, this weight loss was relative, and there was still weight gain compared to the control group. Our findings are consistent with previous reports showing that THC administration reduced the rate of weight gain in rats fed HFD, possibly due to inhibition of fat mass gain (30-33). While THC has been shown to increase appetite and subsequent weight gain in some studies, our findings contrast with this (34). This discrepancy may be explained by the low dose of THC administered in our study. Furthermore, unlike synthetic cannabinoids, natural cannabis has been reported to improve insulin sensitivity, promote weight loss in HFD-induced obese rats, and lower blood sugar levels (14, 18, 26, 31, 32, 34, 35).

Also, it has been demonstrated that long-term administration of THC does not lead to weight gain (30, 31, 34). Furthermore, our results indicated that low-dose THC improved glucose tolerance during the oral glucose tolerance test (OGTT) and increased insulin sensitivity. Despite THC being known to increase appetite, it has been associated with a reduced risk of obesity and, consequently, insulin resistance (14, 18, 31, 32, 35, 36). A recent cross-sectional study also reported that cannabis consumption was linked to lower fasting insulin levels and a reduced risk of insulin resistance, as evidenced by lower HOMA-IR values (36). Additionally, the correlation between cannabis use and lower insulin resistance has been found to be statistically significant compared to non-users (37). The protective mechanism of THC against elevated blood sugar levels and insulin resistance is partially attributed to its role as a potent anti-oxidant against ROS. Reduction of ROS levels significantly improves insulin sensitivity (38).

THC has demonstrated anti-oxidant properties similar to vitamins E and C, enabling it to scavenge free radicals, reduce metal ions, and protect against oxidative processes (39), as confirmed by our present results. The unsaturated bonds present in the non-olivetolic components of THC have been identified as responsible for its anti-oxidant properties (39). These observations indicate that THC exerts extensive protective effects against oxidative stress (OS). In our study, THC was found to modulate the redox balance by increasing the activities of superoxide dismutase (SOD), glutathione peroxidase (GPX), and catalase (CAT), while suppressing malondialdehyde (MDA) production. Additionally, ROS levels were found to decrease in PCOS rats treated with THC compared to untreated PCOS rats. These findings highlight the anti-oxidant properties of THC, which support the findings of previous studies. In this context, it was shown that both the endocannabinoid system (ECS) and peroxisome proliferator-activated receptors (PPARs) could have a substantial impact on PCOS and its associated conditions. This is particularly evident in disruptions of glucose-lipid metabolism, as well as in issues related to obesity and fertility (16). The results of the current study provided evidence of inflammation, as reflected by significant increases in circulating levels of C-reactive protein (CRP) and mRNA expression of TNF-α, IL-6, IL-1β, and MCP-1. Recent studies have shown that immunocompetent cells in the blood of PCOS patients with infertility *in vitro* produce several cytokines, including IFN-γ, TNF-α, and IL-2, which may be involved in chronic inflammation (1-3, 11, 27-29, 40, 41). THC has been demonstrated to possess anti-inflammatory properties by suppressing cytokine production, inhibiting Th1 cells, and activating Th2 cells. Furthermore, THC has been shown to suppress pro-inflammatory cytokines such as IL-1α, IL-1β, IL-6, and TNF-α (41), which is consistent with the present findings. The comprehensive study of these genes aims to elucidate their individual contributions to the intricate inflammatory mechanisms observed in PCOS. Their roles in macrophage activation, cytokine production, and immune regulation collectively contribute to the inflammatory milieu associated with PCOS. Incorporating this information into the research findings will provide a clearer understanding of the molecular pathways involved in PCOS-related inflammation. Each of these genes plays a crucial role in the inflammatory processes associated with PCOS. For example, Cd163 and F4/80 are macrophage markers linked to immune response regulation, while Mcp1 is involved in monocyte recruitment and inflammation. Tnf, Il6, and Il1β are pro-inflammatory cytokines associated with the inflammatory cascade, and Il10 serves an anti-inflammatory role, potentially modulating inflammation. Irf5 is implicated in immune response regulation.

 In the experimental PCOS rat model, treatment with THC led to a reduction in relative mRNA expression of IL-1β, TNF-α, and IL-6, while the relative mRNA expression of anti-inflammatory genes Irf5 and IL-10 increased. These results align with previous studies demonstrating that THC reduces the levels of pro-inflammatory cytokines like TNF-α, interferon-c cytokine, and GM-CSF (granulocyte-macrophage colony-stimulating factor) and down-regulates the expression levels of IL-1α, IL-1β, and IL-6 (41, 42). Additionally, previous studies have indicated that THC improves intestinal inflammation in mouse colitis models (43). Thus, THC shows promise in reducing inflammation in a rat model of PCOS.

Macrophages (M1 and M2) play critical roles in various physiological and pathological processes, including tissue growth, immune response to pathogens, inflammatory reactions, and clearance of aged and apoptotic cells (4-6). Our results demonstrated that THC inhibits M1 macrophage polarization and induces an M2 macrophage phenotype in a PCOS rat model. This finding supports the role of THC in suppressing the inflammatory response and provides evidence for further research on its therapeutic potential. The ratio of M1 and M2 macrophages indicates the nature of each process. Imbalances in the ratio of M1 and M2 macrophages have been reported in pathological conditions and are considered key factors in determining the inflammatory state. During the initial inflammatory phase, the M0 macrophage phenotype transitions to M1-activated macrophages or rapidly polarizes towards the M1 phenotype, leading to the release of large amounts of pro-inflammatory cytokines that ultimately enhance the inflammatory response (4, 25).

But then, macrophages partially transition from M1 polarization to an M2 phenotype to produce anti-inflammatory factors and aid in tissue repair (4, 5). It has been reported that an imbalance in macrophage function disrupts ovulation (4, 6, 25). In this study, our results indicated the presence of M1 and M2 macrophages in rat ovarian tissue, while the induction of PCOS disrupted the M1/M2 ratio. We found that mRNA expression of Mcp1 and F4/80 significantly increased in the THC-treated rat model of PCOS compared to the untreated group. Additionally, flow cytometry results showed a significantly higher percentage of F4/80 and CD206 cells in the THC-treated groups compared to the untreated PCOS group, while the percentage of CD11 cells significantly decreased, confirming the switch from M1 to M2 macrophage polarization. Therefore, THC treatment can restore the balance of the M1/M2 ratio and reduce inflammatory responses in the ovaries, leading to the resumption of normal follicle growth.

Pro-inflammatory M1 macrophages inhibit insulin sensitivity by producing cytokines, while anti-inflammatory M2 macrophages have the opposite effect (25, 42, 44). Our study demonstrated that mRNA levels of M1-associated cytokines such as Mcp-1 were increased in rat ovarian tissue, whereas mRNA of the M2-associated cytokine F4/80 was suppressed in the rat PCOS model. These results were further confirmed by flow cytometry analysis of different types of macrophages in ovarian tissue, where we observed an increased percentage of CD11 cells and decreased percentages of CD206 and F4/80 cells in the PCOS rat model. However, treatment with THC increased the percentage of CD206 and F4/80 cells while decreasing the percentage of CD11 cells. Therefore, it can be speculated that THC attenuates inflammation in the PCOS rat model by influencing the M1/M2 macrophage ratio.

## Conclusion

The results of the present study demonstrate the anti-oxidant and anti-inflammatory properties of THC in HFD/Letrozole-induced PCOS rats. Administration of a low dose of THC ameliorates the metabolic, endocrine, hormonal, and morphological changes in the PCOS rat model. Furthermore, THC reduces insulin resistance in the PCOS rat model by modulating macrophage M1/M2 polarization. THC shows potential as a therapeutic agent against insulin resistance in PCOS. Importantly, the results of this study warrant future investigations into the molecular processes underlying the protective effects of low-dose THC on PCOS-associated metabolic dysfunction and its related metabolic/endocrine complications.

## Authors’ Contributions

FM S performed the experiments, meticulously executing the planned procedures, ensuring the accurate collection of data. S Z contributed to the inception and design of the experiments, demonstrating a profound understanding of techniques. Additionally, S Z authored the paper, crafting a comprehensive and insightful manuscript that presented the research findings and their implications coherently. M N diligently analyzed the data, employing sophisticated statistical methods to draw meaningful conclusions from the experimental results. His expertise in data analysis provided valuable insights into the observed trends and correlations. H HM contributed by providing essential reagents, materials, and analysis tools that were crucial to the successful execution of the experiments. His expertise and support significantly enhanced the experimental process.

## Funding

This research did not receive any specific grant from funding agencies.

## Conflicts of Interest

The authors declare that no conflicts of interest could be perceived as prejudicing the impartiality of the research reported.
